# Optimization of Semiautomated Calibration Algorithm of Multichannel Electrotactile Feedback for Myoelectric Hand Prosthesis

**DOI:** 10.1155/2019/9298758

**Published:** 2019-03-14

**Authors:** Milica Isaković, Jovana Malešević, Thierry Keller, Miloš Kostić, Matija Štrbac

**Affiliations:** ^1^University of Belgrade, School of Electrical Engineering, Belgrade, Serbia; ^2^Tecnalia Serbia Ltd, Belgrade, Serbia; ^3^University of Belgrade, Belgrade, Serbia; ^4^Tecnalia Research & Innovation, San Sebastian, Spain

## Abstract

The main drawback of the commercially available myoelectric hand prostheses is the absence of somatosensory feedback. We recently developed a feedback interface for multiple degrees of freedom myoelectric prosthesis that allows proprioceptive and sensory information (i.e., grasping force) to be transmitted to the wearer instantaneously. High information bandwidth is achieved through intelligent control of spatiotemporal distribution of electrical pulses over a custom-designed electrode array. As electrotactile sensations are location-dependent and the developed interface requires that electrical stimuli are perceived to be of the same intensity on all locations, a calibration procedure is of high importance. The aim of this study was to gain more insight into the calibration procedure and optimize this process by leveraging a priori knowledge. For this purpose, we conducted a study with 9 able-bodied subjects performing 10 sessions of the array electrode calibration. Based on the collected data, we optimized and simplified the calibration procedure by adapting the initial (baseline) amplitude values in the calibration algorithm. The results suggest there is an individual pattern of stimulation amplitudes across 16 electrode pads for each subject, which is not affected by the initial amplitudes. Moreover, the number of user actions performed and the time needed for the calibration procedure are significantly reduced by the proposed methodology.

## 1. Introduction

Humans rely on their hands to grasp, manipulate objects, and carry out a variety of activities of daily living. Tactile feedback is one of the key components that enable dexterous use of hands [1]. Hand amputation and loss of these essential functions are traumatic events leaving to dramatic consequences on everyday life. Research and technological advancement in prosthetic hands resulted in commercial devices that today range from simple grippers with one degree of freedom (DOF) to dexterous robotic hands that support multiple DOFs and grasping configurations [2]. The most technologically advanced noninvasive technique to partially restore the functions of the missing hand is by employing myoelectric prosthesis. These systems can recognize user intentions from electrical activity of remaining muscles (EMG) [3]. By employing the muscles originally used to accomplish the desired tasks, the user can intuitively operate the artificial hand [4]. This holds a great promise to improving the quality of life for hand amputees, which is why significant research efforts are aimed at further optimizing existing solutions [5] and providing a more intuitive user control [6]. However, the user will find a limited benefit from these improvements when using multi-DOF prosthesis, if somatosensory feedback, which is crucial for effective motor planning and execution [7, 8] in grasping and object manipulation tasks [9], is missing.

Including feedback and closing the loop in prosthetic systems are important goals pursued by the researchers over the last four decades [10] and have also been acknowledged from the user's perspective as the most wanted improvement [11]. It needs to be noted that the advantages the feedback provides are not limited to the aspect of control but that benefits can also be found in reduction of phantom-limb pain [12] and sensations of prosthesis embodiment [13].

Natural, somatotopically matched sensory feedback can be delivered to amputees invasively, via direct nerve [14–16] or brain [17] stimulation. Another approach to close the loop is known as sensory substitution [18]. In this method, the data are read from the prosthesis sensors and this information is transmitted to the user through a controlled activation of his/her preserved sensory systems. The feedback can be delivered noninvasively through vibro- [19] or electrotactile [20] skin stimulation. In the latter, low-level electrical current pulses are delivered to the skin to depolarize superficial afferents and elicit tactile sensation. The intensity and quality of sensation, and thereby the information content, can be regulated by changing the stimulation parameters (i.e., pulse width, amplitude, and/or frequency coding) and the location of the stimulation delivery (i.e., spatial coding) [20].

Over the past decade, our group's research efforts have been focused on leveraging the multipad electrode technology to deliver the high-quality proprioceptive and interaction force feedback in an intuitive manner [21–26]. The developed approach relies on the dynamic stimulation patterns, where messages are coded using frequency and spatial modulation (i.e., changing the stimulation frequency and location of the active electrode pad), to communicate the state of the prosthesis in an intuitive manner [22]. Recently published results show that this approach enables precise communication of the prosthesis state [22] and that it has a steep learning curve, enabling intuitive use that does not additionally burden the user [25].

A drawback of electrotactile stimulation, amplified in multipad (array) systems, is the variability of elicited sensations. Even though sensitivity to electrotactile stimulation has some topological regularity [21], when observed on the level of precision needed for sensory substitution, significant intrasubject and temporal variability is observed [21, 22, 27, 28]. An additional concern is the significant overlap of preferred and uncomfortable amplitude ranges between different subjects as described in [21]. This implies that an a priori set value that is in the preferred range for most users could cause unpleasant sensations in some of them. To overcome this and avoid any discomfort, a sequential calibration procedure should be applied in every session.

As described in our previous work [22–26], the method of limits [29] is an adequate procedure, where sequential scanning of sensations in a predefined amplitude range is iteratively performed for each pad until clear sensations of similar intensity were observed throughout the electrode. Over the course of our research, this procedure was streamlined to the point where it would rarely take more than 5 minutes, which was well within the acceptable range for the experimental setup [22]. However, for everyday use of such technology, this cumbersome setup procedure would be a strong deterrent for users and could significantly impede the adoption.

In this paper, we present the results of research effort to simplify the system personalisation and calibration process. First, the question of electrotactile variability is addressed in more detail to confirm the need for calibration and to determine possible strategies for algorithm simplification. Later, we investigate benefits of two different approaches identified based on statistical analysis of the gathered data.

## 2. Materials and Methods

### 2.1. System Setup

The system setup included a wireless multichannel electrotactile stimulation system (MAXSENS, Tecnalia Research & Innovation, San Sebastian, ES) and a laptop PC (Intel® Core™ i5-4210U CPU at 1.70 GHz, 6 GB RAM) running MATLAB (R2016a, The MathWorks, Natick, MA) application with GUI for semiautomated calibration of stimulation intensity.

**Figure 1:**
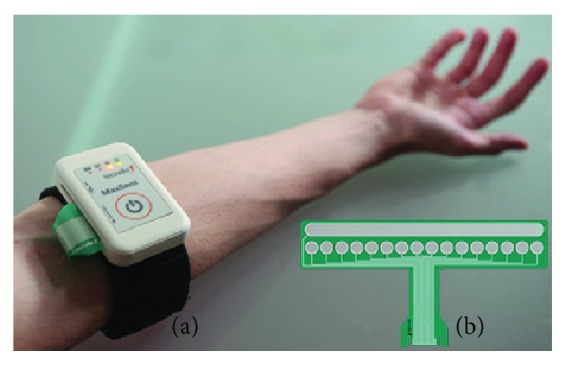
(a) The stimulation system, comprising the stimulation unit and the array electrode inside the brace with an adjustable strap, positioned on the forearm. (b) The stimulation array electrode with 16 circular cathodes and a common anode.

The stimulation system, presented in Figure 1, is a fully programmable and integrated multichannel interface comprising a stimulation unit and a flexible array electrode [22]. The stimulation unit generates current-controlled biphasic stimulation pulses, with parameters suitable for the electrotactile stimulation (pulse width from 50 to 1000 *μ*s with a 10 *μ*s step, pulse rate from 1 to 400 Hz with a 1 Hz step, and amplitude from 0.1 to 5 mA with a 0.1 mA step). The unit is equipped with a Bluetooth communication interface, allowing control of the stimulation parameters and active channels from the host PC using a simple set of commands. The stimulation array electrode, with 16 circular pads (cathodes) and a common adjacent anode, was designed to be placed circumferentially around the forearm. It was custom-designed and made on a 125 *μ*m PET substrate using Ag/AgCl conductive paste and an insulation coating for biomedical applications covering the conductive leads. The pads were covered with conductive hydrogel (AG735, Axelgaard, DK) in order to improve the contact between the electrode and the skin.

### 2.2. Protocol

Nine able-bodied subjects (4 female, 5 male, age 29 ± 5 years, all right-handed) gave their informed consent and participated in the study. The subjects were comfortably seated in front of the table with the laptop PC. The electrode was positioned circumferentially around the subject's left forearm, 5 cm below the elbow. It was positioned by ensuring that the two middle pads are centered on the middle of the volar side (see Figure 1(a)).

The electrode was positioned at the beginning and removed at the end of each session. Each session was followed by at least 30 minutes of pause. Before the beginning of the first session, each subject was introduced with an explanation of the calibration procedure and had the opportunity to familiarise with the GUI used. Subjects were instructed to calibrate the stimulation intensity with a goal of obtaining similar sensations for all pads, sensations that are distinct, but pleasant, and to ensure that there is clear spatial separation between the adjacent pads. An important difference in respect to the previous studies [22–27] where the calibration procedure was managed by the expert researchers is that here the calibration was performed by the lay subjects without any assistance.

Each subject participated in 10 sessions of *standard calibration procedure*, performed throughout 3 days. The standard calibration procedure, which we previously used in a study with able-bodied and amputee subjects [22], includes 2 phases.

**Figure 2:**
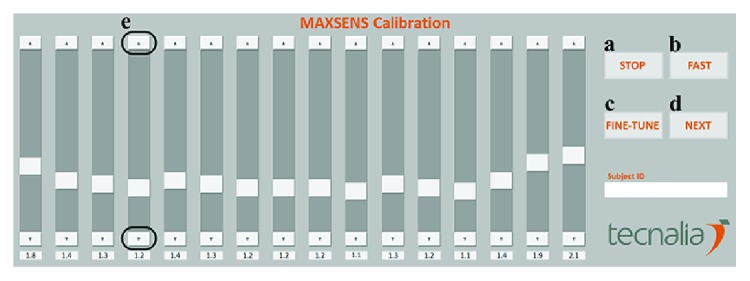
Calibration GUI implemented in MATLAB. (a) STOP button used in phase 1 of the standard calibration procedure for stopping the increase in stimulation intensity when a satisfying sensation is reached. (b) FAST button for activation of fast scanning sequence of previously selected intensities. (c) FINE-TUNE button used to start the fine-tuning protocol. (d) NEXT button used for transition to the following pad if necessary. (e) “up” and “down” arrows for adjusting the amplitude of the corresponding pad during fine-tuning.

In phase 1, the PC application automatically increases the stimulation amplitude of the first pad starting from 1 mA with a 0.1 mA step until the subject indicates to have perceived a pleasant, but distinct sensation by clicking the appropriate button in GUI (Figure 2(a), STOP button). This is repeated for each of the 16 pads of the electrode. When phase 1 is finished, the subject is stimulated with every pad with the selected intensities in a fast scanning sequence. By clicking the FAST button (Figure 2(b)), each pad is activated for 0.2 seconds, starting from pad no. 1 and moving circumferentially to pad no. 16. This enables the subjects to quickly feel transitions between the pads and test if the perceived sensations are indeed similar for all pads.

Phase 2 of the standard calibration procedure is aimed at adjusting the baseline amplitudes obtained in phase 1 in a simple and systematic manner. To allow subjects to identify subtle differences in sensations between adjacent pads and fine-tune the amplitude, each pad is activated before and after the previous pad, as well as before and after the following pad. As an example, part of the sequence for fine-tuning pad no. 5 and no. 6 is 5-4-5-6-5-6-7-6. In this sequence, each pad (except the first and last ones) is activated 3 times for 2 seconds, so the whole fine-tuning process lasts 92 seconds. The subject fine-tunes the intensities for each pad by clicking the “up” and “down” arrows on the corresponding slider (Figure 2(e)). At the end of the procedure, the subject is once again presented with calibrated intensities for all pads. Fine-tuning (phase 2) can be repeated if the subject is not satisfied with the intensities, i.e., if the sensations of the same intensity are not perceived for each pad of the electrode.

After all subjects completed 10 sessions of standard calibration procedures, the protocol was modified to simplify and accelerate the calibration procedure. Instead of starting from 1 mA and obtaining the baseline amplitudes through the phase 1 procedure, we leveraged the knowledge gained from the results of previous calibration sessions to set a priori values for all pads. Here, following the logic outlined in the discussion, we decided to set the baseline amplitudes of all pads at the 25th percentile value of all 90 standard calibration sessions. To test if in this way similar results can be obtained, on the fourth day each subject performed two additional sessions using this *streamlined calibration procedure*.

### 2.3. Data Analysis

Calibration curves obtained in all sessions of standard and streamlined calibration procedures were visually inspected.

The coefficient of variation (CV), also known as relative standard deviation (RSD), was calculated to examine the dispersion of amplitudes for each subject and every pad. It is expressed as a percentage and defined as the ratio of the standard deviation to the absolute mean value.

In order to explore individual patterns of the curves which occur in all subjects, we applied correlation analysis. Correlations between each of the 10 calibration curves and their individual mean curve from 10 sessions, as well as the overall mean curve for all sessions and subjects, were calculated and averaged for each subject. The validity of the streamlined calibration procedure was confirmed through correlation of the obtained calibration curve and the mean curve from the standard procedure. For each subject, we also calculated the correlation between the baseline curve (overall 25th percentile) and the final calibration curve in the streamlined calibration procedure. Paired-samples *t*-test was used to compare calculated correlation coefficients.

For each of the 90 selected amplitude curves (10 sessions × 9 subjects), we calculated the total distance measured in mA, from 3 possible starting curves:

1. Default constant used in the standard calibration process, i.e., 1 mA for each pad
2. Optimal constant, calculated as the value that results in the smallest difference from all calibration curves, i.e., 1.8 mA
3. Mean curve for all subjects and sessions

The total distance was calculated as the sum of absolute differences between two values for all pads. Statistically significant differences between 3 baseline curves were assessed using a one-way repeated measure ANOVA with Greenhouse-Geisser correction, followed by a post hoc pairwise comparison with Bonferroni correction.

## 3. Results and Discussion

**Figure 3:**
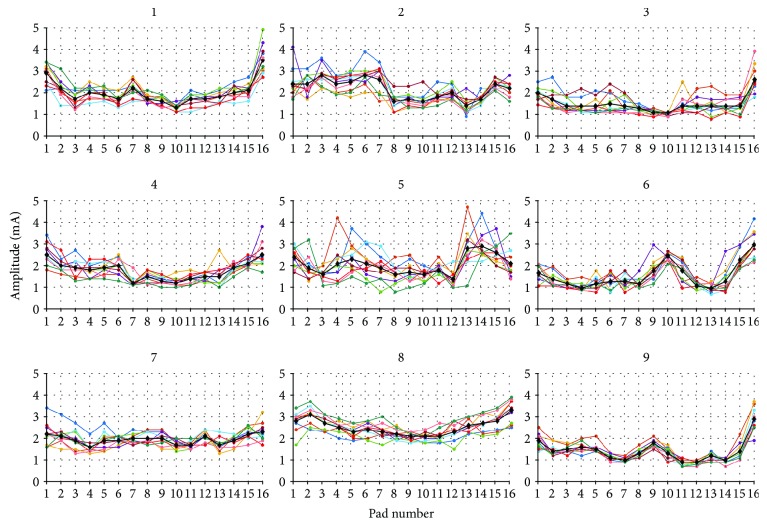
Amplitude values (vertical axis) for every electrode pad (horizontal axis) obtained through 10 sessions of calibration for each of 9 subjects. Individual measurements are presented through coloured lines, while the overall means are presented with a black line.

To examine intersubject and intrasubject variability of preferred stimulation amplitudes through the electrode array, we analysed the data of 10 standard calibration sessions for individual subjects. The resulting preferred amplitudes obtained in this process are presented in Figure 3 for all 9 subjects. Each panel contains stimulation amplitudes for 16 pads obtained during 10 calibration sessions (coloured lines) and their mean value (black line).

The coefficient of variation (in %) for each subject and for every pad is presented in Table 1. On average, the largest variance of 26.6% was noted in subject 5, while the smallest variance (13.1%) is in the measurements of the subject 8.

Despite the variance, distinctive individual patterns can be observed in visual inspection of the results presented in Figure 3.

Correlation coefficients between each measurement and the mean for each subject were calculated to confirm the existence of these patterns. As shown in the second column of Table 2, the high correlation values confirm the existence of individual patterns. Such result concurs with [21] confirming existence of individual electrotactile maps and are also in the accordance with the findings described in [28], describing temporal variability of sensitivity to electrical stimulation.

To determine if a general rule can be deduced from these results, we also analysed the group values. The correlations of individual subject measurements with the overall mean curve were calculated, as shown in the first column of Table 2.

These correlations are significantly smaller in comparison with those calculated with respect to the individual average (paired-samples *t*-test, *p* = 0.0033). However, for five of nine subjects they are larger than 0.65, which encouraged us to further investigate the possibility of generalisation.

**Figure 4:**
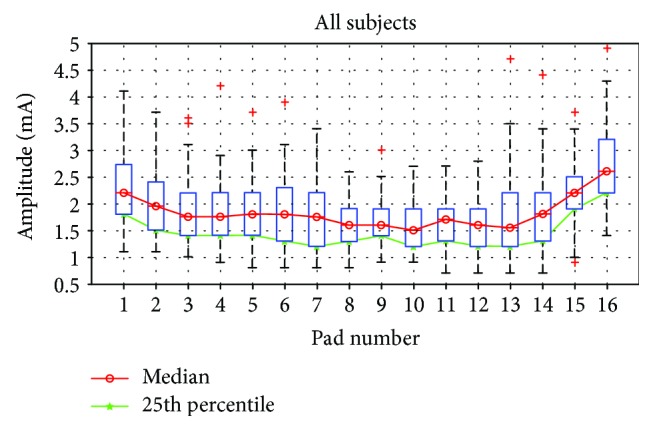
Boxplots of amplitudes for 16 pads and all subjects. Median value and 25th percentile of all data are presented with red and green line, respectively.

A boxplot of selected stimulation amplitudes for all calibration sessions and all subjects (10 × 9) are presented in Figure 4. On visual inspection, this curve shows some of the characteristic features noted in all individual patterns. The most obvious one is the convex shape, which again is in accordance with [21] and can be explained by the anatomical features. As shown in [21], the volar side of the forearm is more sensitive than the dorsal, and a similar relation was noted between the medial and lateral sides. Furthermore, this may explain the peak intrasubject variability (Table 1) observed in the pads that may shift from the dorsal to volar side based on the minor change in the electrode placement, i.e., 4-5 medial and 13-14 lateral (depending on the forearm size).

In all calibration sessions, the time to reach the baseline was above three minutes, and the results were such that the fine-tuning procedure was always required. This motivated us to determine a priori baseline values that are similar enough to the desired result that it can be achieved through the fine-tuning procedure only. As suggested by the results shown in the first column of Table 2, the overall mean could present a good candidate for the *a priori* baseline. The second candidate we considered was the optimal constant, calculated as the value that results in the smallest difference from all selected amplitudes.

**Figure 5:**
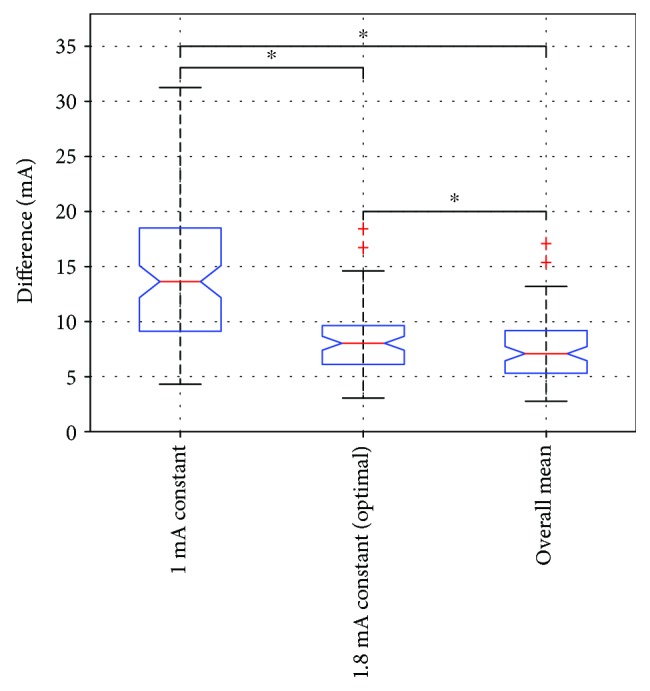
Boxplots of distance from 3 different starting curves for all calibration curves. Horizontal bar with asterisks indicates statistically significant difference in mean difference from the calibration curves between the respective conditions.

To estimate the efficacy of the process depending on the baseline value, the amount of user actions needed to achieve the preferred stimulation amplitudes was calculated. This was defined as the cumulative “distance” in mA between the starting and selected amplitudes. The comparison was made between the two selected candidates, as well as with the baseline used in the *standard calibration* process, which was 1 mA. The results are presented in Figure 5.

A repeated measures ANOVA with a Greenhouse-Geisser correction determined that the mean difference between the selected values and the starting values differed statistically significantly between 3 different options (*F*(1.038, 92.372) = 113.639, *p* < 0.0001). Post hoc tests using the Bonferroni correction were also performed. The differences obtained with optimal constant were significantly lower compared to the 1 mA starting line (*p* < 0.0001). The tests revealed that the overall mean starting curve resulted in smaller differences from the calibration curves (7.43 ± 2.75 mA), compared to both the 1 mA constant (14.23 ± 6.04 mA) and the optimal 1.8 mA constant (8.19 ± 2.80 mA), which were both statistically significant (*p* < 0.0001).

These results suggest that use of the overall mean as the baseline value would present an efficient solution. However, the values on the mean curve are above the range of selected amplitudes on more than one pad for three subjects (4 pads for subject 3, 7 pads for subject 6, and 8 pads for subject 9). Earlier experience [22–25] suggests that subjects prefer starting with lower intensity and reaching the desired value by increasing the intensity, than the other way around. Furthermore, as higher amplitudes lead to faster habituation [19, 20], a procedure that seeks the minimal acceptable value is more appealing than the alternative. Since the overall data were normally distributed (Anderson-Darling test, *p* < 0.05 for all 16 pads), and therefore mean and median calibration curves are very close, we decided to consider other descriptive statistics such as quartiles and percentiles.

When this additional constrain was applied, the 25th percentile (i.e., lower quartile) curve became an obvious candidate for the baseline in the *streamlined calibration procedure*. These values are within or below the range of selected amplitudes for each subject and are strongly correlated with the mean curve.

**Figure 6:**
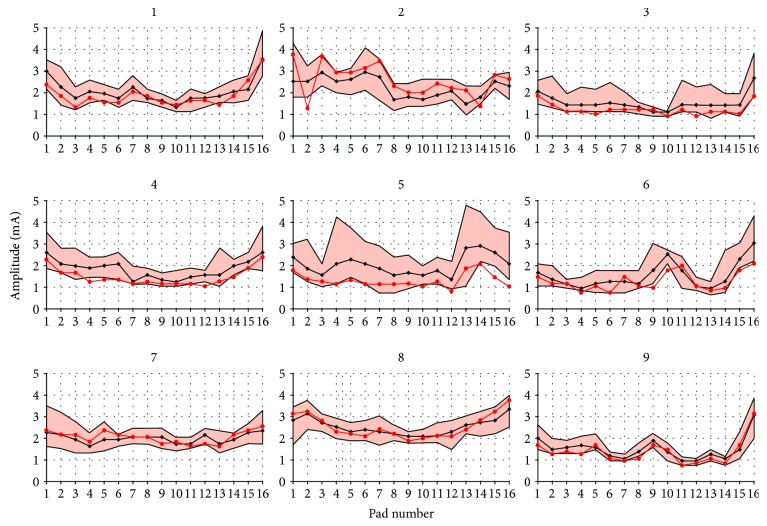
Results of the fine-tuning calibration (red line) compared to the results of the 10 calibration sessions (range— shaded area, mean—black line).

As noted before, the streamlined procedure comprised only phase 2. Results of this calibration process are presented in Figure 6. We can note that the values obtained in this way are within the range of the values obtained with the standard calibration procedure and that the obtained curve follows a similar trend. This is confirmed by high values of correlation coefficients between the curve obtained in the optimized calibration session and the mean of 10 sessions, shown in Table 2. These range from 0.66 to 0.96, with an average of 0.84 ± 0.10. These are systematically higher than the correlation between the selected values and the baseline (25th percentile), shown in the last column of Table 2, suggesting that such starting point did not significantly influence the selection of amplitude values.

All subjects managed to perform the calibration procedure in a single sweep, meaning that the theoretical minimum of 92 seconds was reached. Furthermore, subjects 2, 5, and 6 noted that this procedure was lengthy, and in the second *streamlined* session opted not to use the fine-tuning procedure but several iterations of the “fast” mode (Figure 2(b)), where after quick scanning of all electrode pads they could correct the amplitudes where they detected an uneven sensation. As this was not foreseen in the experimental protocol and the time of execution was not measured, this cannot be reported in detail. However, it showcases the potential usability of multipad electrotactile technology, as well as of the proposed method.

The study was conducted in able-bodied subjects, which allowed unambiguous positioning of the electrode array by measuring the distance from the elbow and ensuring that the two middle pads are centered on the middle of the volar side of the forearm. It should be noted that the procedure for electrode placement would be to a certain degree more complex for amputees, since it must also consider some additional factors like length of the stump, presence of neuroma, and problems with skin sensibility. However, based on previous experience from several studies in patients with amputation [22, 23, 25, 26], small variations in electrode position that were introduced due to these factors did not affect calibration results or increase the time required for electrode calibration.

The presented method of calibration was designed for the electrotactile stimulation system with the 16-pad electrode array positioned circumferentially around the forearm, intended to be incorporated in the prosthesis socket and present feedback from myoelectric prosthesis to the user. However, immediate results, i.e., calibration curves, are not relevant only for this single application, but can be used as reference values when setting amplitudes of any type of electrotactile display that is positioned on the forearm. As an example, these functions can be remapped to electrodes intended to convey feedback information in teleoperation or virtual reality gaming. Moreover, an indirect result of this study is the verification that adaptation of baseline in respect to electrode location can shorten and simplify the calibration procedure. Therefore, this same approach for identification of baseline values can be applied in electrotactile displays independent of electrode configuration and position. These systems can be used for transmission of realistic tactile sensation in many emerging application fields, such as providing feedback in lower-limb prostheses [30], vestibular substitution [31], assistive devices for the visually impaired applied to various body parts (back [32], tongue [33], forehead [34], fingertips [35], or palm [36]), virtual reality and telexistence [37], and touch panels with haptic feedback [38].

## 4. Conclusions

A novel method for calibration of the multipad electrotactile sensory substitution system was presented. The method comprises a sequential algorithm supported by an intuitive GUI that allowed 9 lay subjects to perform the calibration process in the time comparable to that reported when performed by experts. Results show that with use of a priori knowledge in setting the baseline stimulation amplitudes, this process can be significantly streamlined, to a quarter of initial time, without affecting the quality of the outcome.

Based on these findings, we hypothesize that further improvements can be achieved by additionally leveraging a posteriori knowledge in subsequent calibration sessions, i.e., by choosing personalised baseline values informed by the previous selections by that specific subject. This will be the subject of future investigation.

## Figures and Tables

**Table 1:** Coefficient of variation (in %) of selected amplitudes in the 10 initial sessions.

Pad	1	2	3	4	5	6	7	8	9	10	11	12	13	14	15	16	Mean ± STD
Subject																	
1	17	20	20	15	13	16	17	10	15	12	18	13	13	15	15	20	15.6 ± 3.0
2	29	20	18	14	17	18	23	23	19	23	16	16	28	14	8	15	18.8 ± 5.4
3	15	26	20	25	27	31	22	12	13	7	28	23	30	21	23	24	21.7 ± 6.8
4	19	16	23	18	17	20	20	17	14	16	17	12	34	11	11	24	18.1 ± 5.7
5	20	30	18	44	29	30	32	28	29	17	17	26	34	22	20	30	26.6 ± 7.3
6	23	25	12	19	23	31	23	20	28	8	28	16	18	44	19	21	22.4 ± 8.2
7	25	19	23	16	23	10	11	13	15	12	9	12	17	12	13	19	15.6 ± 5.1
8	17	14	11	10	12	14	15	11	9	10	13	18	11	15	14	16	13.1 ± 2.7
9	18	19	13	15	14	12	10	14	10	20	21	11	15	11	26	18	15.4 ± 4.5

**Table 2:** Comparison of correlation coefficients and the results of statistical analysis.

Subject number	Correlation between 10 calibration curves and mean curve from all sessions (mean ± STD)	Correlation between 10 calibration curves and individual mean curve from 10 sessions (mean ± STD)	Correlation between streamlined calibration curve and individual mean curve from 10 sessions	Correlation between streamlined calibration curve and overall 25th percentile curve
1	0.86 ± 0.04	0.92 ± 0.05	0.90	0.88
2	0.35 ± 0.18	0.85 ± 0.10	0.68	0.23
3	0.69 ± 0.07	0.78 ± 0.10	0.84	0.69
4	0.76 ± 0.16	0.84 ± 0.10	0.93	0.90
5	0.24 ± 0.29	0.69 ± 0.15	0.87	0.05
6	0.47 ± 0.12	0.88 ± 0.05	0.83	0.52
7	0.40 ± 0.20	0.61 ± 0.15	0.66	0.74
8	0.74 ± 0.16	0.87 ± 0.11	0.95	0.83
9	0.74 ± 0.08	0.94 ± 0.06	0.96	0.85
Mean ± STD	0.59 ± 0.22	0.82 ± 0.11	0.84 ± 0.10	0.63 ± 0.31
Paired-samples *t*-test	*p* = 0.0033 (significant)		*p* = 0.4502 (nonsignificant)	*p* = 0.0483 (significant)

## Data Availability

The data used to support the findings of this study are available from the corresponding author upon request.
